# Caring for Young People Who Self-Harm: A Review of Perspectives from Families and Young People

**DOI:** 10.3390/ijerph15050950

**Published:** 2018-05-10

**Authors:** Sophie Curtis, Pinar Thorn, Alison McRoberts, Sarah Hetrick, Simon Rice, Jo Robinson

**Affiliations:** 1 Orygen, The National Centre of Excellence in Youth Mental Health, Parkville, VIC 3052, Australia; sophie.curtis@orygen.org.au (S.C.); pinar.thorn@orygen.org.au (P.T.); alison.mcroberts@orygen.org.au (A.M.); simon.rice@orygen.org.au (S.R.); 2 Centre for Youth Mental Health, The University of Melbourne, Parkville, VIC 3010, Australia; s.hetrick@auckland.ac.nz; 3 Department of Psychological Medicine, Faculty of Health and Medical Sciences, The University of Auckland, Auckland 1010, New Zealand

**Keywords:** young people, self-harm, families, parents, carers, support, resources

## Abstract

Self-harm among young people remains largely stigmatised and misunderstood. Parents have been identified as key facilitators in the help-seeking process, yet they typically report feeling ill-equipped to support the young person in their care. The aim of this review was to examine the perspectives of both young people (aged 12–28) and parents and to develop the conceptual framework for a future qualitative study. A systematic search of MEDLINE and PsycINFO was performed to identify articles that focused on the experiences of family members and young people related to managing the discovery of self-harm. Fourteen articles were included for review. Four addressed the perspectives of young people and 10 reported on the impact of adolescent self-harm on parents. The impact of self-harm is substantial and there exists a discrepancy between the most common parental responses and the preferences of young people. In addition, parents are often reluctant to seek help for themselves due to feelings of shame and guilt. This highlights the need for accessible resources that seek to alleviate parents’ distress, influence the strategies implemented to manage the young person’s self-harm behaviour, reduce self-blame of family members, and increase the likelihood of parental help seeking.

## 1. Introduction

Self-harm is defined as any form of intentional self-injury or self-poisoning, irrespective of motive or suicidal intent. Self-harm is associated with an increased risk of mental health problems, suicide, and other adverse outcomes, such as poor educational results and premature death due to risk-taking behaviour [1,2]. Whilst these behaviours are linked to a heightened risk of suicide, not all people who engage in self-harm are suicidal. In these instances, self-harm may occur in an attempt to cope with, or to regulate, intense emotional or psychological distress [1,2]. Given that global self-harm rates have increased over the past two decades [3,4,5], self-harm remains a priority public health concern.

Adolescence represents a critical period of development during which substantial neurological and biological changes occur. In conjunction with these changes, adolescents are exposed to new challenges related to study and work, romantic relationships, and increasing responsibility and independence [6]. As a result of the complex interplay between genetic, biological, psychiatric, psychosocial, and cultural influences, self-harm often begins during adolescence, with the onset of self-harm commonly associated with the beginning of puberty [7,8].

### 1.1. Prevalence of Self-Harm in Young People

Obtaining information regarding the prevalence of self-harm is problematic given that most young people who engage in self-harm do not seek professional help [9]. Furthermore, whilst hospital admission data provide an indication of self-harm rates and trends, these reveal only the tip of the iceberg as the majority of people who self-harm do not require medical attention, and many hospital presentations do not lead to admissions [10].

Despite the above, community prevalence estimates of self-harm can be inferred based on self-report data. Whilst global variation exists, community research suggests that around 10% of young people have engaged in self-harm behaviours [11,12,13,14,15,16]. In most countries self-harm occurs more frequently in females than males, with a sex ratio as high as 6:1 between the ages of 12 and 15 years [2]. Furthermore, repeated self-harm is common in adolescents with almost two thirds of those who have self-harmed reporting doing so more than four times [11].

In addition to young females, other populations of young people experience disproportionately high rates of self-harm. For example, young people who identify as LGBTIQA+ are twice as likely to engage in self-harm, and this is often the result of bullying and discrimination [17,18,19,20]. Similarly, within Australia, young people aged 15–24 years of Aboriginal or Torres Strait Islander descent are up to five times more likely than non-indigenous youths to engage in self-harming behaviours [21]. This discrepancy is widened by the overrepresentation of Aboriginal or Torres Strait Islander young people in juvenile justice facilities, where approximately 80% of people have a diagnosed mental illness [22]. The rates of self-harm are higher in clinical samples, particularly those with depression, anxiety [23], substance use [16], and borderline personality disorder (BPD) [24]. An awareness of these subgroups of young people, and the unique challenges they face, is essential when addressing their complex support needs, particularly in the context of self-harm.

### 1.2. Help-Seeking Among Young People

Help-seeking includes seeking support from formal service providers (e.g., health professionals) as well as informal connections (e.g., family members and peers). As mentioned above, only a minority of young people who engage in self-harm seek help [9]. This is concerning given that early intervention and prevention programs have been found to reduce the severity of physical injuries incurred as a result of self-harm, as well as lower the risk of future suicide attempts [25].

Numerous barriers to help-seeking amongst young people have been identified. Fears related to confidentiality breaches, stigma, being appraised as “attention seeking”, and receiving negative reactions are common interpersonal barriers to help-seeking [9,26,27,28]. In addition, the presence of depression, anxiety, and suicidal ideation, as well as a belief that one should be able to cope on one’s own, further hinders the ability to reach out [27,29]. Conversely, assurances of confidentiality, being treated respectfully, having a trustworthy person to talk to, and the option of talking to someone of a similar age and background have been identified as facilitators to help-seeking behaviour [28,30].

The literature suggests that of those young people who do seek help, the majority turn to informal supports [9,26]. Whilst peers remain the preferred source of support amongst young people [30,31], parents have been identified by young people as key facilitators in the help-seeking process [26,30]. Indeed, evidence suggests that connectedness between young people and their parents and other carers serves as a protective function for a range of health-outcomes among young people [32,33]. The initial response of parents has been found to affect the timing of formal help-seeking among young people [34] and influence the likelihood of future help-seeking [28]. Unfortunately; however, parents remain uncertain regarding how best to support and manage a young person who is engaging in self-harm [30], do not initiate the professional help-seeking process until the young person experiences a number of difficulties independent of self-harm [34], and are often perceived by young people as responding harmfully [1,30]. While parents may feel uncertainty, when they are appropriately informed and supported (e.g., provided with parenting and family skills training, parent education, and social support), they can play an instrumental role in initiating treatment and supporting young people who self-harm. Notably, efficacious psychosocial treatments for self-harm thoughts and behaviors for young people focus on treating the family, as well as the young person [35]. The global increase in self-harm rates amongst young people raises questions related to the availability and utility of resources designed to provide support to both young people and their parents and carers.

Therefore, the aim of this narrative review is to analyse the common themes from both the perspective of young people and parents and carers. This review will advance understanding of both perspectives, inform consultation exercises with young people and their families, and inform a qualitative study that will adapt an existing resource for parents and carers.

## 2. Materials and Methods

### Search Strategy

The literature search involved an investigation of the experiences of family members and young people with regards to managing the discovery of self-harm within the family. A systematic search of the electronic databases MEDLINE and PsycINFO was performed in September 2017. The following search terms formed the basis of the search strategy: (Parent* OR Carer* OR “Support Person” OR Adult* OR Relative* OR Relation OR Guardian OR “Family Relation*” OR Caregiver* OR “Family Member*” OR Family) AND (“Young Person” OR “Young People” OR “Young Adult” OR Teen* OR Adolesc* OR Youth* OR Student*) AND (“Suicidal Ideation” OR “Self Harm*” OR “Self Injurious Behaviour” OR “Self Mutilation” OR “Self Inflicted Wounds” OR “Self-poison*” OR “Self-injur*” OR “Self-cut*”) AND (Support* OR Resource* OR Program* OR Group OR Brochure OR Pamphlet OR Guideline* OR “Support Group*” OR “Written Communication”). Reference lists of key review papers and included articles were also searched. The search was restricted to academic literature published between 2007 and 2017.

From the pool of potential articles, those that were included for full review and data extraction contained primary research data and specifically addressed: (1) opinions of young people (age range 12–28) on how best to help young people who self-harm and their experience of support after self-harm disclosure; or; (2) parental experiences of supporting a young person through self-harm. Articles were excluded if they were a secondary source, or related to medication, were explicitly related to specific mental health disorders (e.g., BPD), related to self-harm in other populations (i.e., older adults), focused on discussion of screening measures, prevalence or risk factors or were specific to mental health professionals.

## 3. Results

**Figure 1:**
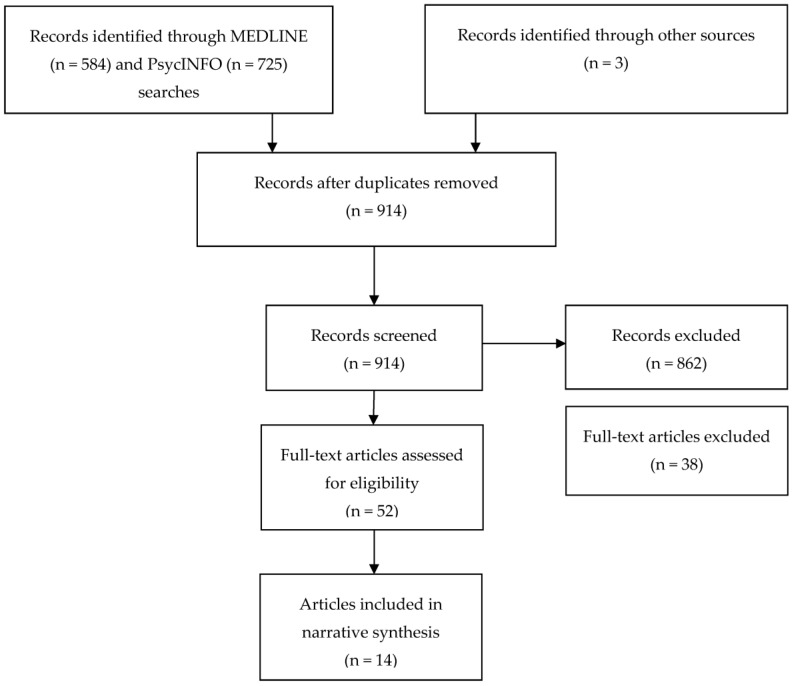
Search flow diagram.

From the search, 1312 articles were identified, of which 14 were included for this review. The literature search is summarised in Figure 1 below.

Of the included articles, four addressed the perspectives of young people with regards to how best to help young people who self-harm or their experience of support after self-harm disclosure (primarily to parents and peers). Of those four articles, three reported data generated from one study. Ten articles examined the impact of adolescent self-harm on parents and carers. Of those 10 articles, three articles used the same dataset to answer different research questions. All of the articles related to perspectives of young people involved quantitative analysis, whilst those addressing parental perspectives were qualitatively analysed. Studies were conducted across Australia, the UK, USA, and Ireland. A summary of the included articles is displayed in Table 1 and Table 2 and a narrative synthesis of the findings is presented below.

### 3.1. The Perspectives of Young People

One common theme amongst the articles examining young people’s perspectives was the report that emotionally charged reactions from parents and other caregivers and disciplinary measures are unhelpful and often detrimental [1,30]. These responses, combined with a parental fear of stigma and a general reluctance to seek professional help, have been found to intensify distress in the young person and promote self-harming behaviour as a coping strategy [30]. Conversely, young people reported that the most helpful strategies for assisting them in managing their self-harm were talking and listening (i.e., maintaining open communication) in a non-judgmental way [1,26,30,31]. More specifically, studies identified that parents can help by remaining calm, acknowledging the intensity of their child’s distress, directly asking the young person for guidance in how they can assist, and openly offering their time [1,31].

In addition to talking and listening, young people indicated that parents could support them by providing referrals to professional services and other adults who might be able to help [1,26,30,31]. Active parenting such as gathering family members together for pleasurable activities or to resolve problems, and encouraging the young person to seek help from school staff, are examples that have been provided regarding informal referrals [26,30].

Less frequently suggested, though still important are the views that parents should refer their child to professionals including counsellors, psychologists, GPs or psychiatrists [1,26,30]. The endorsement of helping a young person by referring them to someone, including an adult, relative or teacher, supports previous suggestions that adults should take action when they recognise self-harm [30].

Finally, young people expressed their concerns regarding stigma and confidentiality as barriers to help-seeking [1,26,30,31]. Fears related to judgement and labelling from adults may increase the tendency to turn to peers for support over parents [31]. In addition, young people feel that assurances of confidentiality increase feelings of trust and thus the likelihood of self-harm disclosure [1,26]. In order to address this, they recommend that parents and carers remain non-judgmental and respect the young person’s privacy as far as possible [1,26,30].

Table 3 summarises the above and categorises young people’s views on parental support in the context of self-harm into five broad themes identified by Berger, et al. [30]. The content related to three family sub-themes (more love, talk to family members, and family problems) identified by Fortune, et al. [26] have also been incorporated in the table.

### 3.2. The Perspectives of Parents and Carers

Ten of the included articles investigated the impact that self-harm by young people can have on parents and carers. The results indicated that in addition to the detrimental effects that self-harm has on the young person, it also has a significant impact on their parents and carers [34,36,37,38,39,40,41,42,43,44].

Caregiver strain can be conceptualised as the adverse thoughts, feelings, and consequences experienced by those caring for someone who is suffering [44]. Despite little research in this area, the general consensus is that parental well-being and functioning are directly impacted by child mental health status. This was highlighted by all of the 10 included articles that addressed the perspectives of parents and carers [34,36,37,38,39,40,41,42,43,44]. Specifically, parents of young people who self-harm reported experiencing significant feelings of guilt and shame, doubts about their ability to parent effectively, fears of exacerbating self-harm and lack of social support and resources to guide them in supporting their young person [37,38,40,42,43,44]. Ferrey and colleagues [38], found that the impact of self-harm discovery on family members is widespread, extending beyond the immediate psychological consequences. This was supported by several of the other articles and included: initial and ongoing impacts on mental health, impact on partners, siblings and wider family networks, social isolation, work and financial complications, and parental perceptions of the future [36,37,38,39,41].

The discovery of self-harm can also lead to changes in parenting strategies and has been shown to alter the power structure in the family as parents become fearful of imposing boundaries for their child [39]. The negative emotions and uncertainty experienced by parents in response to self-harm may lead them to disengage from discussing their child’s behaviour or, alternatively, attempt to exert control [36,37]. For example, parents have reported reading their child’s emails and diaries following the discovery of self-harming behaviour in order to monitor their emotional state [39,41]. These common reactions are those that young people believed to be the most unhelpful [30]. On reflection however, parents also reported that these intense emotional reactions can be detrimental to the parent-child relationship and that instead, maintaining open communication and demonstrating care, affection or love are much more helpful responses [37,39].

Parents typically find communication with their child about self-harm to be a difficult process [40,41]. Kelada and colleagues [41] revealed that parents feel uncertain about how to address the topic and doubt their abilities to support their young person. Parents in this study tended to describe their child is terms such as “unapproachable” and stated that they felt ill-equipped and fearful that discussions could trigger another episode of self-harm [41]. Despite these feelings of uncertainty and the indication that parents require assistance in managing and relating to their child, the stigma attached to self-harm often means that parents are reluctant to seek information and help to support them in their caring role [37]. This can result in perceived isolation, making it difficult for parents to access information that could help them to cope and support their child. Parents have therefore expressed a need for practical and easily accessible information and support, including written information, that may assist them in managing their young person’s self-harm [43].

Bryne, et al. [31] identified parents and carers require both support and information to meet their internal (e.g., emotions and beliefs) and external (e.g., knowledge and coping strategies) needs. In Table 4 the relevant information above has been summarized and presented in one of these two categories.

## 4. Discussion

This review identified 14 articles that had a primary focus on the experiences of family members and young people with regards to managing the discovery of self-harm within the family. Of the 14 included articles, 10 of them reported on the perspectives of parents compared to only four that focused on the views of young people. The findings highlight that the impact of self-harm is substantial, and that the wellbeing of parents and carers can be significantly impacted. In addition, there exists a disparity between the most common parental responses to self-harm and the preferences of young people. For example, attempts to exert control, heightened monitoring and disciplinary measures are perceived by young people to be unhelpful and can serve to perpetuate their self-harming behaviours [41,45]. It was also noted that in many cases, young people prefer to talk to their friends, as opposed to their parents. However parents can play an important role if provided with appropriate support and information.

Understanding the ways in which parents make sense of their child’s self-harm is imperative, as it has been shown that the conceptualisation of self-harm can influence the strategies implemented to manage the behaviour [39]. For example, when parents attribute their child’s self-harm to their developmental stage, this often leads to the use of more supportive parenting strategies [39]. The same is true when parents understand self-harm in the context of mental health difficulties [39]. However, when self-harm is perceived as “naughty”, “bad” or an attempt to exert control, this can result in counterproductive responses such as dismissive disengagement or increased monitoring [39]. This suggests that providing education to parents regarding the broad range of factors that render a young person vulnerable to self-harm, could result in more accurate conceptualisations of the behaviour, and thus the implementation of more supportive and effective parenting strategies.

Given that parents report feeling substantially ill-equipped and isolated when attempting to manage a young person’s self-harm, the results of the present review support the need for more open communication and easily accessible resources that seek to alleviate parents’ distress and isolation. Indeed, parents from the study conducted by Stewart and colleagues [43] expressed a need for practical resources (including leaflets and web resources). Parents are often reluctant to seek help (formal or informal) as a result of the immense feelings of shame and guilt that they experience in relation to their child’s self-harm [37]. This is particularly problematic, as young people indicated that it would be helpful for their parents to facilitate connections with informal supports, such as teachers [30]. Moreover, sometimes disclosure of a young person engaging self-harm is made to parents by school staff [34]. This highlights that the importance of building supportive communities and training gatekeepers, such as school staff—see Robinson, et al. [46] for a review of school-based interventions. Despite their reluctance to seek help, parents frequently indicate that they require support in order to manage their child’s behaviour [37]. This suggests that reducing the stigma attached to self-harm is an essential step in order to promote help seeking behaviour among parents and reduce the incidence of self-harm in young people. In the meantime, given that the internet is used as a resource for health information [47], there is a need for increased availability of online resources that parents can access from the privacy of their own home. This could enable them to contextualise self-harm, avoid self-blame, improve the quality of their relationship with their child, implement effective support for their child, and increase their likelihood of seeking help and support for themselves and their child.

Finally, the divergent views held by young people and by parents identified by the current review, suggest the clear need for information, resources, and models of care and support that can balance the needs of both groups, in order that both young people and their parents/carers receive optimal support. Such resources or treatment strategies should be developed in consultation with young people and parents, delivered, and rigorously evaluated in order to ensure that they are both acceptable and effective.

### Limitations

When considering the findings of this review, several limitations need to be taken into account. Firstly, whilst the search strategy was thorough, it is possible that some articles may have been missed, particularly those outside of the specified date range or in languages other than English. Secondly, there is currently no standardised definition of self-harm, and therefore the term may have been employed non-systematically across studies, making it difficult to compare findings. Thirdly, while the articles included were based on large samples of adolescents recruited through schools, the vast majority of young people did not have personal experience of thoughts of self-harm or an actual episode of self-harm, thus their perspectives may differ from young people with lived experience of self-harm. Furthermore, the quantitative and open-ended survey questions employed in the two separate studies did not permit in-depth exploration of the topic or further elaboration of responses. As a result, researchers might not have been able to comprehensively and thoroughly answer research questions. Finally, this was not a systematic review, and as such no double screening or data extraction were conducted; nor is an assessment of study quality provided. That said, it is noted that a number of the included studies were conducted in Western countries and did not employ random sampling procedures, and thus the generalisability of the results in this review may be limited. Overall, given that the present review took the form of a narrative synthesis, results should be treated as an overview, not an expansive, systematic presentation of the evidence.

## 5. Conclusions

Despite the above limitations, this review offers an important insight into the experiences and views of parents and carers who are supporting a young person with self-harm.

Self-harm among young people is a significant public health issue, and one which remains largely stigmatised and misunderstood. It is estimated that one in 11 young people between the ages of 12 and 17 have self-harmed at some point in their life, yet worryingly, less than a half of these seek help [9,11]. When help is sought, peers tend to be the preferred source of support, reflecting the increased need for independence from parents during this developmental period [30,31]. However, turning to peers for support may also reflect the perception that parents lack understanding and may not be equipped to help [31]. Indeed, research has consistently indicated that parents feel uncertain and fearful when faced with a child who is engaging in self-harm and experience intense feelings of guilt and shame [36,41]. As such, providing evidence-based information about self-harm to parents could increase their confidence in managing and supporting their young person.

Dissemination of this information is likely to assist in the reduction of feelings such as guilt and shame, as well as the stigma that serves as a significant barrier to help-seeking for both parents and young people. Researchers, clinicians and policy makers can assist this process by making information about self-harm more widely available in a range of formats, thus educating the public and reducing misconceptions. It is imperative however, that resources of this nature continue to be informed by the experiences, needs and perspectives of both parents and young people. Empowering parents to effectively support a young person through self-harm is essential for the prevention of self-harm repetition, promotion of future help-seeking, and reduction of suicide risk.

## Figures and Tables

**Table 1:** Articles that examined the perspectives of young people (*n* = 4).

Study	Aims	Description	Results	Limitations	Implications
Berger, et al. [30]. *(Australia)*	To explore adolescent views of what parents and teachers can do to assist young people who self-harm.	A school-based sample (*n* = 2637; aged 12–18 years) completed a self- report questionnaire.	Females were more likely than males to suggest parents and teachers talk with young people, and to comment on the importance of family relationships, while those with a history of self-harm were less likely to suggest communication and more likely to reflect on the role of family conflict. Other responses included: referring to another adult who could help, enlisting professional help, and ensuring confidentiality.	Participants were not randomly selected and therefore the sample was potentially biased by parents of young people who self-harm withholding their consent from participation due to the sensitive nature of the topic. School absentees could have also been biased to self-harm. As a result, findings may not be generalised to other settings or circumstances.	Listening and providing support is not only the role of professional. Parents and teachers have a large part to play. This highlights the need for tailored resources that educate parents and teachers regarding communication about self-harm.
Berger, et al. [1]. *(Australia)*	To identify adolescent perspectives on how peers and online friends can assist young people who self-harm, and examine differences according to age, gender, and exposure to self-harm.	Students (*n* = 2637; aged 12–18 years) from 41 schools completed survey questions asking them to describe what peers and online friends could do to help young people who self-harm.	Talking and listening was the most common response. Females were more likely to suggest referral to someone else who had self-harmed, or a reliable adult. Males were more likely to feel as though no one could help. There was a general ambivalence towards seeking help online. Younger participants were more inclined to talk to young people and refer to adults, whereas older participants were more concerned about stigma and pessimistic about available help.	Representativeness of the sample may be limited due to participants being self-selecting. In addition, key terms were not defined for participants and may have been used interchangeably.	Adolescents, particularly young males, require information and education that assists them to identify, and safely respond to, peers who self-harm. Findings also highlight the reluctance to seek help from adults. To be effective, educational programs addressing self-harm need to be evaluated by adolescents.
Fortune, et al. [26]. *(UK)*	To identify what adolescents believe can be done to prevent self-harm urges, and to investigate differing views according to gender, ethnicity, and previous self-harm behaviour.	Students (*n* = 6020; aged 15–16 years) from 41 schools completed a self-report questionnaire.	Eleven categories of responses were identified. These related to the causes of self-harm and what can be done i.e., talk/listen, families, activities, friendship and peer interactions, school, formal organisations, barriers to seeking help, substances, public education, media, difficult to prevent. Family, peers, and schools were more salient than formal organisations.	Only half of the sample responded to the question related to self-harm prevention, and there were differences between those who responded and those who did not. Young people who had dropped out of school did not participate and this group may be biased by a greater tendency to self-harm.	Supporting a healthy network of family and peer relationships should underpin government policies. Furthermore, school counsellors are required in all schools. School-based mental health programmes should be provided for all pupils and anti-bullying interventions in schools are also required.
Hasking, et al. [31]. *(Australia)*	To explore the impact of self-harm disclosure by young people over time.	A sample of 2637 adolescents (*n* = 2637; aged 12–18 years) completed self-report questionnaires at three time points, one year apart.	Among young people who had self-harmed, 59% reported disclosure of self-harm to someone else. Those who disclosed were more likely to have friends who also self-harmed, and they reported that they had previously sought help for emotional issues. Disclosure facilitated help-seeking from peers, improved coping and reduced suicidality. Confiding in an adult may be a protective factor, as youth who reported disclosing to adults also reported better psychosocial functioning over time.	The people who were on the receiving end of disclosure were not mutually exclusive so the effect of disclosure specifically to adults or friends on future functioning remains uncertain. Furthermore, the measures of disclosure and help-seeking may be confounded, as disclosure of self-harm may be perceived as a form of help-seeking.	Information and support initiatives should be directed primarily to the young person’s peer group and to parents as the two priority groups. Parents and friends of young people who self-harm should also be encouraged to model the use of problem-focused coping strategies.

**Table 2:** Articles that examined the perspectives of parents and carers (*n* = 10).

Study	Aims	Description	Results	Limitations	Implications
Byrne, et al. [36]. *(Ireland)*	To describe parents’ and carers’ experiences of self-harm in their child in order to identify their support needs.	Parents (*n* = 15) and carers (*n* = 10) of young people who had engaged in self-harm or had expressed suicidal ideation attended a focus group.	Significant difficulties in family communication, parent-child relationships and discipline were described. The need for peer support, psycho-education, communication and parenting skills, re-establishing family structures, and practical support for handling self-harm incidents, were expressed.	Due to recruitment from services, findings may not generalise accurately to parents whose children have not attended services. Individual interviews would have provided information above and beyond that disclosed in the groups.	Findings highlight areas and issues that health professionals need to address in order to offer appropriate help and support to parents/carers. In light of findings, an eight-week group programme has been developed and is being evaluated.
Ferrey, et al. [37]. *(UK)*	To respond to parents needs by creating a website for parents containing video/audio clips as well as transcripts from interviews with the parents.	Interviews were conducted with parents and carers (*n* = 39) of young people aged up to 25 years who had self-harmed.	Discovery of a child’s self-harm resulted in feelings of shock, powerlessness and an absence of control. One response to these feelings was to exert control. Some parents had positive experiences with clinical services whereas others did not. Parents found it difficult to speak with others who had not been through a similar situation.	Only the experiences of parents were collected (not those of young people) and no sample demographics were displayed.	Parents hearing the experiences of other people in the same situation can function as a source of information and form of virtual support.
Ferrey, et al. [38]. *(UK)*	To explore how and why parenting changes after the discovery of self-harm.	Semi-structured narrative interviews were conducted with parents (*n* = 37) of 35 young people who had self-harmed.	Parenting strategies were altered after the discovery of a young person’s self-harm. These changes included: increased or decreased support, control, and monitoring of the child.	There were few fathers who took part and thus, results reflect predominantly the views of mothers. In addition, diversity was limited in the sample and the views of young people were not investigated.	Clinicians and school staff with responsibility for young people should assist parents to find strategies that are effective for their child and themselves. This could include providing psychoeducation about the nature of self-harm, and a discussion of possible parenting strategies to manage it.
Ferrey, et al. [39]. *(UK)*	To explore the effects of self-harm on parents and family and to create information that could assist parents in providing care.	In-depth interviews were conducted with parents (*n* = 37) of 35 young people under the age of 25 who had self-harmed.	The effect of a young person’s self-harm on parents extends beyond emotional states to include mental health, relationships with others, work and finances. Parents required information that may help them understand and manage self-harm.	Sample diversity was limited and only opinions of parents were gathered. Thus, authors could only report on their interpretation of the impact on their children and family members.	Formal agencies should be aware of the impact self-harm may have on parents. Parents are in need of information about self-harm. Support groups may serve the dual purpose of information and social support.
Hughes, et al. [40]. *(UK)*	To explore parental experiences of adolescent self-harm and how they make sense of the behaviour.	Narrative interviews were conducted with parents and other family members (*n* = 41) of 38 young people, aged up to 25, who had self-harmed.	The primary response of parents was to try to make sense of their child’s self-harm. This involved three sequential processes: (1) initial reactions of bewilderment and confusion; (2) search of information and (3) attempts to build a new way of seeing.	Most participants were mothers and ethnic diversity was limited. Different perspectives on sense-making may have been apparent in a more diverse sample.	Professionals can support parents to make sense of self-harm behaviour by showing understanding, listening to their perspectives, and making them aware of the broad range of factors than can lead to self-harm. Information about self-harm needs to be made readily available and more easily accessible.
Kelada, et al. [41]. *(Australia & USA)*	To assess the impact self-harm has on parent health, parenting and interactions with professional help.	Study 1: Australian parents (*n* = 16) of adolescents with a history of self-harm responded to open-ended questions about their child’s self-harm. Study 2: American parents (*n* = 22) of adolescents with a history of self-harm participated in interviews.	Parents experienced emotional and psychological reactions to self-harm. They underestimated the frequency of, and were not well informed about, their child’s self-harm. Intense emotional reactions from parents were detrimental to adolescent behaviour, yet communication was positive. Shifts in parenting were noted following disclosure of self-harm and parents felt ill-equipped to deal with the situation. Professionals remaining empathic was key in promoting help-seeking.	Differing timelines of self-harm were present in the sample of parents and retrospective recall may have effected responses. Only parental perceptions were obtained, not those of young people. A large portion of the sample had a history of mental health issues and different methodologies were used in the American versus Australian studies.	Treatment involving families may offer positive prognoses and interventions should aim to empower parents by addressing their lack of knowledge, the impact on parental wellbeing, the potential shift in power dynamics and how best to respond.
McDonald, et al. [42]. *(Australia)*	To examine the experiences of mothers dealing with self-harming adolescents and to gather insights about the ways that this impacts their own well-being and that of their families.	Mothers (*n* = 6) of young people who were self-harming or had a history of self-harm participated in conversational interviews, which allowed them to describe their experience.	The primary response in mothers was feelings of guilt and shame and these feelings worked to isolate them from support networks. Mothers were caring and extremely concerned rather than neglectful. In addition, mothers revealed that they had searched for a sensible understanding of the self-harming phenomenon; to attach some meaning to it for themselves and their child.	The study involved a very small sample size. The views of minority groups were not captured and given that participants were self-selected families where abuse had occurred may have been less likely to participate.	Family health workers need to normalize the experience of guilt and shame. They can give parents information about the motivating factors of self-harm and discuss options for helpful strategies to manage within the family. They can also provide psychoeducation on coping skills and affirm that it is appropriate for them to seek help
Oldershaw, et al. [34]. *(UK)*	To explore perspectives of parents related to service provision, making sense of self-harm, and its impacts.	Parents (*n* = 12) of adolescents referred to Child and Adolescent Mental Health Services CAMHS for treatment of self-harm engaged in semi-structured interviews.	Parents were aware of self-harm before external agencies but tended to view it as a phase and resist help initially. Parents also struggled to make sense of self-harm and advised others to seek help sooner than they had done. Input from external agencies and offers of help affected the timing of help-seeking.	Sample limited to two CAMHS teams. Only half of the parents approached agreed to participate and thus findings may be biased.	Parents may benefit if the time from discovery of self-harm to referral is reduced and this may be facilitated via guidance from external agencies. The mere offering of support may be more valuable than its content or provision.
Stewart, et al. [43]. *(UK)*	To explore parents’ experiences of treatment and support for young people and for themselves in the context of self-harm.	Semi-structured narrative interviews were conducted with parents (*n* =37) of 35 young people (aged up to 25 years) who had self-harmed at any point in the past.	Parents felt unprepared for the process of caring for a young person following self-harm and appreciated support to help them navigate this unfamiliar world. A major theme was the need for professionals to have the right attitude. Parents also talked about practical aspects of treatment and the need to have access to help early on. Parents wished to be involved in their child’s treatment and to be listened to.	Lack of ethnic diversity, dominance of mothers, and the opinions of young people were not explored.	Attitudes towards young people who self-harm can make a considerable difference to engagement and motivation. Public health should consider making appropriate information regarding self-harm more widely available, for instance, through leaflets, websites and booklets.
Whitlock, et al. [44]. *(USA)*	To examine the effects self-harm on parents by analysing differences in - caregiver strain between caregivers of youth with self-harm and those of youth without self-harm.	Participants were parents of young people who self-harm (*n* = 196) and parents of young people who do not self-harm (*n* = 57). All completed self-report questionnaires.	Parents of young people who self-harm face increased stress particularly concerning self-blame, regret or guilt. Parent perceptions of responsibility for the behaviour contributes to these feelings. Compassion for self and child was inversely related to both objective and subjective internal stress. Internal stress was significantly influenced by feeling supported in social networks.	Lack of random sampling techniques limit the generalisability of the results. Furthermore, given there was no group of parents of youth with mental health concerns but no self-harm, it is unclear whether self-harm in youth contributed uniquely to parental stress.	Parents of young people who self-harm experience secondary stress that impacts functioning and well-being. Encouraging parents to seek help for themselves may help reduce their levels of stress and improve their ability to manage their child’s behaviour.

**Table 3:** What young people believe parents can do to help young people whom have engaged in self-harm.

Theme	Description
Talk and Listen	Parents should talk to the young person, and actively listen them to try to understand the function of self-harm. Parents should be educated about self-harm so that they can learn how to appropriately communicate with young people about the issue.
Referral/connection to Adults	Parents should alert school personnel, gather family members to solve problems and refer the young person to, or connect them with, other adults that may be able to help.
Formal Organisations	Parents can refer young people to professionals such as counsellors, psychologists or psychiatrists.
Reduce Stigma and Ensure Confidentiality	Parents need to be open-minded and non-judgemental whilst respecting the privacy of the young person.
Family Context	Young people wish for more love, attention, time, support and care from families. Parents should take an active interest in the young person and their life, as well as trying to make the young person happy and understanding the challenges that young people face. Parents should attempt to reduce conflict in the family, avoid emotionally charged reactions, and increase family activities that might distract the young person from their problems. Moreover, young people require physical safety within the home and a stable family environment.

**Table 4:** What parents and carers need to help themselves and the young people whom have engaged in self-harm.

Theme	Description
Support	Parents and carers require prompt and adequate support and guidance from mental health services, health professionals, and schools. It is important for parents to be involved in their child’s treatment plans. Parents would benefit from professionals actively listening to their perspectives, acknowledging their feelings and normalising their feelings of confusion, guilt, and shame. Moreover, professionals could take an individualised approach to helping parents in a way that fits their lifestyle. It is important for parents to take care of themselves and acknowledge their own needs. Parents would benefit from obtaining professional help for themselves to cope with their own distress and challenges they are faced with, as well as manage the situation and support their child. Parents would benefit from both social support (e.g., friends and relatives) and peer-support (e.g., individuals or groups with lived experience) to manage the impact self-harm has had on their lives. Peer-support in particular reduces feelings of isolation and functions as a source of information and mutual social support from other parents who have experienced a child’s self-harm, especially in the context of a support group with strangers. Parents would benefit from flexibility in the workplace, where possible (e.g., being able to leave work on short notice). Parents would benefit from interventions that empower them and teach skills and strategies (e.g., how to regulate and appropriately express their own emotions, openly communicate, and adaptively resolve conflict) to understand their role in supporting the young person, improve their parent-child communication, set and maintain boundaries, navigate discipline, manage conflict, and rebuild their relationship with their child. This in turn would increase their confidence in their parenting abilities. Parents require skills and support to improve family communication and rebuild relationships within the family.
Information	Parents and carers require reliable information about mental health and self-harm and what to expect. They also need information about helpful strategies and available treatment options and services to manage and prevent self-harm incidents. Information should be widely available and easily accessible. Information sources such as professionals, other parents, Internet, and traditional paper-based resources are all helpful. Parents and carers require practical advice on how to prevent and manage self-harm episodes. Case studies are helpful.
